# Clinical Features and Outcomes of Treatment for Fourth Nerve Palsy

**Published:** 2010-01

**Authors:** Abbas Bagheri, Mohammad-Reza Fallahi, Mohammad Abrishami, Hossein Salour, Maryam Aletaha

**Affiliations:** Labbafinejad Medical Center, Shahid Beheshti University of Medical Sciences, Tehran, Iran

**Keywords:** Trochlear Nerve Diseases, Strabismus

## Abstract

**Purpose:**

To evaluate the clinical features, etiology and outcomes of treatment for superior oblique (SO) palsy over a 10-year period at Labbafinejad Medical Center.

**Methods:**

A complete ophthalmologic examination with particular attention to forced duction test (FDT) and tendon laxity was performed in all patients preoperatively. The palsy was divided into congenital and acquired types.

**Results:**

Overall, 73 patients including 45 male (61.6%) and 28 female (38.4%) subjects with mean age of 19.7±11.7 (range, 1.5–62) years, were operated from 1997 to 2007. SO palsy was congenital in 56 (76%) and acquired in 17 (24%) cases. The most common chief complaint was ocular deviation (52.1%). FDT was positive in only 7 (9.7%) cases. Other clinical findings included amblyopia (19.2%), head tilt (13.7%), chin down position (4.1%), facial asymmetry (6.8%) and tendon laxity (2.7%). Mean preoperative vertical deviation was 16.1 prism diopters (PD) which was decreased to 1.9 PD postoperatively. Mean exotropia and esotropia were 15 and 13.9 PD respectively before the operation and both decreased to 1.5 PD of horizontal deviation postoperatively. The most common type of SO palsy based on Knapp’s classification was type 3 (42.5%). The most common operated muscle was the inferior oblique (83.6%) and the most common type of operation was inferior oblique myectomy (83.6%). The success rate for initial surgery was 84% and was increased to 96% with a second intervention.

**Conclusion:**

The most common form of SO palsy requiring surgical intervention was congenital which occurred most frequently in young males. Most cases of SO palsy can be successfully treated with a single surgical procedure.

## INTRODUCTION

Although sixth nerve palsy has been reported as the most common type of extraocular muscle palsy in some studies,^1^,^2^ others have noted fourth nerve palsy to be more common in strabismus clinics^3^ while sixth nerve palsy is more prevalent in neuro-ophthalmic clinics.^1^,^4^ In children, the most common category of third and fourth nerve palsies is congenital, while sixth nerve palsy is usually due to infections and immunologic processes.^5^ In adults, however, the most common cause of third and sixth nerve palsy is vascular disorders^6^,^7^, and trauma is the leading cause of fourth nerve involvement^1^,^8^ and other causes are infrequent.^4^–^13^

The force generation test is initially helpful in the diagnosis of paralytic strabismus, but with time, the forced duction test (FDT) becomes positive even long after improvement of the paralysis.^1^,^3^ The goal of treatment in these cases is to improve diplopia and abnormal head posture, and to restore the aesthetic condition of the eyes.^3^ This study evaluates the clinical features, etiology and outcomes of surgery in patients with fourth nerve palsy over a 10-year period at our center.

## METHODS

This retrospective study was performed on hospital records of patients with superior oblique (SO) palsy who were operated at Labbafinejad Medical Center, Tehran, Iran from 1997 to 2007. Patients were followed for at least 6 weeks postoperatively. Data including age, gender, laterality, signs, symptoms, visual acuity, refractive error, deviometry and sensory findings were documented. The surgical procedure and operated muscles were also documented. Findings at 1 week, 1, 3 and 6 months, and final examination were recorded for the purpose of the study. The patients were diagnosed based on findings of the 3-step test, evaluation of ductions, versions and forced duction and force generation tests. The patients were divided into congenital and acquired palsy groups according to history. In cases with unclear etiology, thyroid function test, tensilon test, electromyography and imaging of the orbit and brain (including CT and MRI scans) were performed and if any abnormality was detected, appropriate treatment would be selected. Surgical procedures included ipsilateral superior rectus (SR) recession for Knapp types IV and V when FDT was positive, contralateral inferior rectus (IR) recession for Knapp types II and IV without SO tendon laxity, superior oblique (SO) tucking for Knapp types II and III, and inferior oblique (IO) weakening for Knapp types I and III. The Harada-Ito procedure was reserved for patients with torsional diplopia but no significant vertical deviation in primary position.^1^

## RESULTS

**Figure 1:**
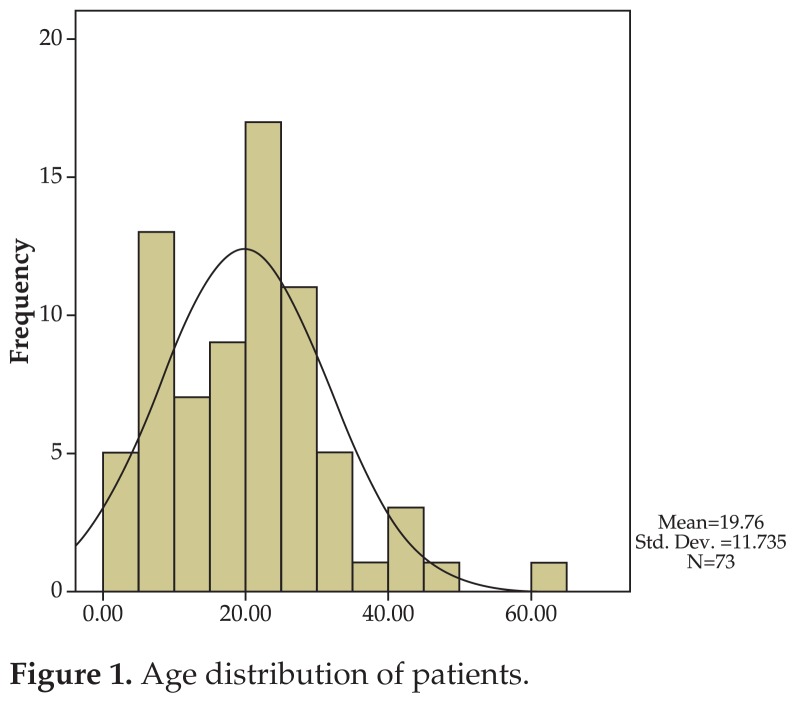
Age distribution of patients.

Overall, 83 patients with superior oblique muscle palsy were operated during a 10-year period. Ten subjects were excluded due to incomplete data. Finally, 73 cases including 45 (61.6%) male and 28 (38.4%) female patients with mean age of 19.7±11.7 (range, 1.5–62) years were studied (Fig. 1). The left eye was affected in 35 (47.9%) patients, the right eye was involved in 28 (38.4%) subjects and the condition was bilateral in 10 (13.7%) individuals.

**Figure 2:**
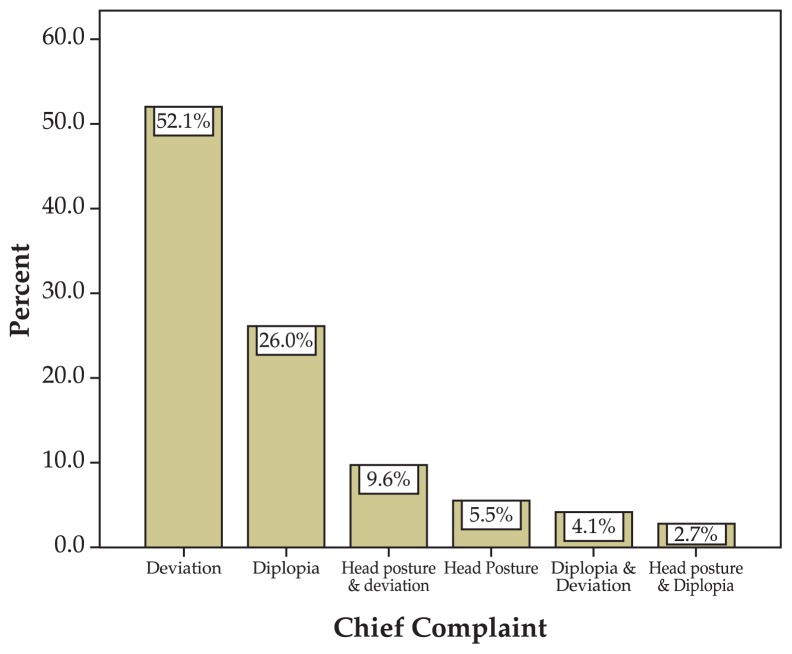
Chief complaints.

**Figure 3:**
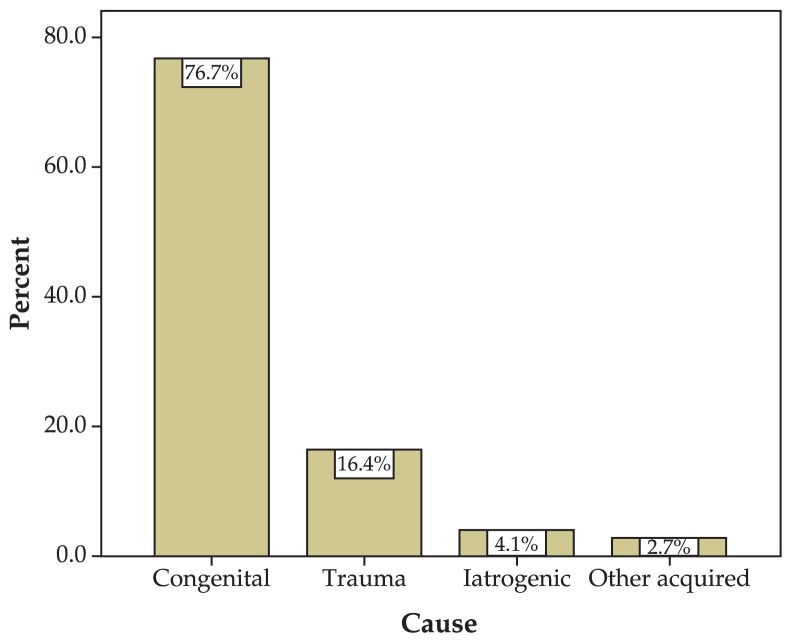
Etiology of SO palsy.

The most common symptom was ocular deviation; diplopia and abnormal head position were less frequent (Fig. 2). No case of face turn was detected. Other findings are summarized in Table 1. Congenital SO palsy was much more frequent (76.7%) than the acquired type (23.3%). In the acquired type, trauma was the most common cause (16.4%) (Fig. 3). Knapp III was the most frequent group according to Knapp’s classification (Table 2).

**Figure 4:**
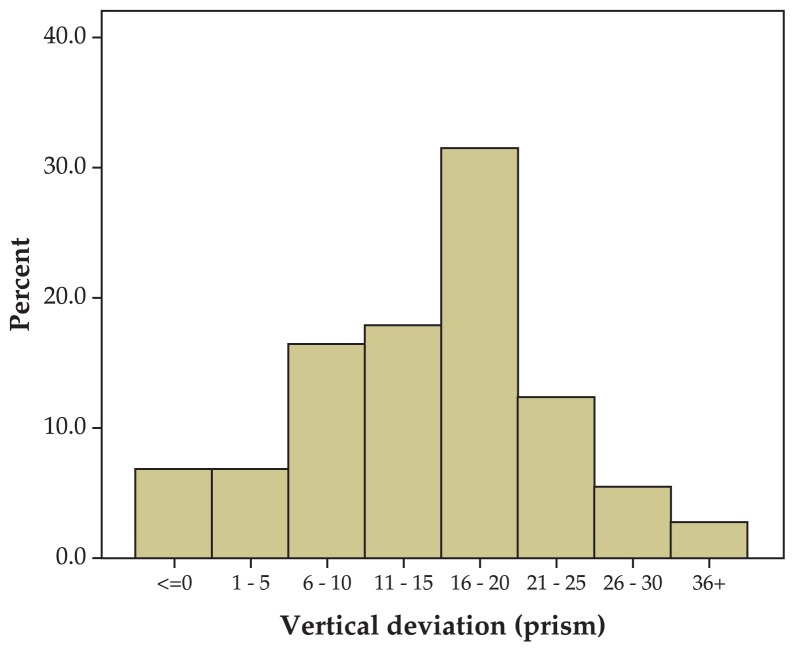
Preoperative vertical deviation.

Fifty two (71.2%) cases were myopic with mean myopia of −0.5±0.3 D and 20 cases (27.4%) were hyperopic with mean hyperopia of +2.3±1.4 D. Mean astigmatism was −0.3±0.1 D overall. Mean vertical deviation was 16.2±8.3 PD (range, 0–40) preoperatively which was decreased to 1.9±4.0 PD (range, 0–6) postoperatively. The mode of vertical deviation in primary position was in the range of 15–20 PD (Fig. 4). There was some degree of inferior oblique overaction in 62 (84.9%) cases and varying amounts of superior oblique underaction in all cases (100%).

Horizontal deviation was present in 48 (65.8%) cases preoperatively including exotropia in 37 (50.7%) patients with mean deviation of 15±9.5 PD, and esotropia in 11 (15.1%) cases with mean deviation of 13.9±11.5 PD. Mean horizontal deviation decreased to 1.5±4.8 PD at final follow-up. Mean decrease in vertical and horizontal deviation after the operation was 14.3 and 13.5 PD respectively (P=0.05).

Acceptable improvement (decreased symptoms and abnormal head position, and eye deviation less than 5 PD) was achieved after one procedure in 61 (83.6%), after two operations in 9 (12.2%), and after 3 times surgery in 2 (2.7%) patients. Only one (1.4%) case required four procedures. The most effective surgery was the first one.

During initial surgery, the most common operation was inferior oblique (IO) weakening performed in 61 (83.6%) cases, and included myectomy in 59 (96.7%) or recession in 2 (3.3%) cases. Other surgical procedures during initial surgery included ipsilateral SR recession in 11 (15.1%), SO tucking in 8 (11%), contralateral IR recession in 8 (11%), the Harada-Ito procedure in 7 (9.6%) and ipsilateral IR resection in one (1.4%) patient. Horizontal muscle surgery was performed during the first operation in 14 (19.2%) patients. Surgical procedures in the second operation included IO weakening in seven, ipsilateral SR recession in four and horizontal muscle surgery in two cases. The third operation was two cases of repeat IO myectomy. The only case of fourth surgery consisted of IO extirpation-denervation.

**Figure 5:**
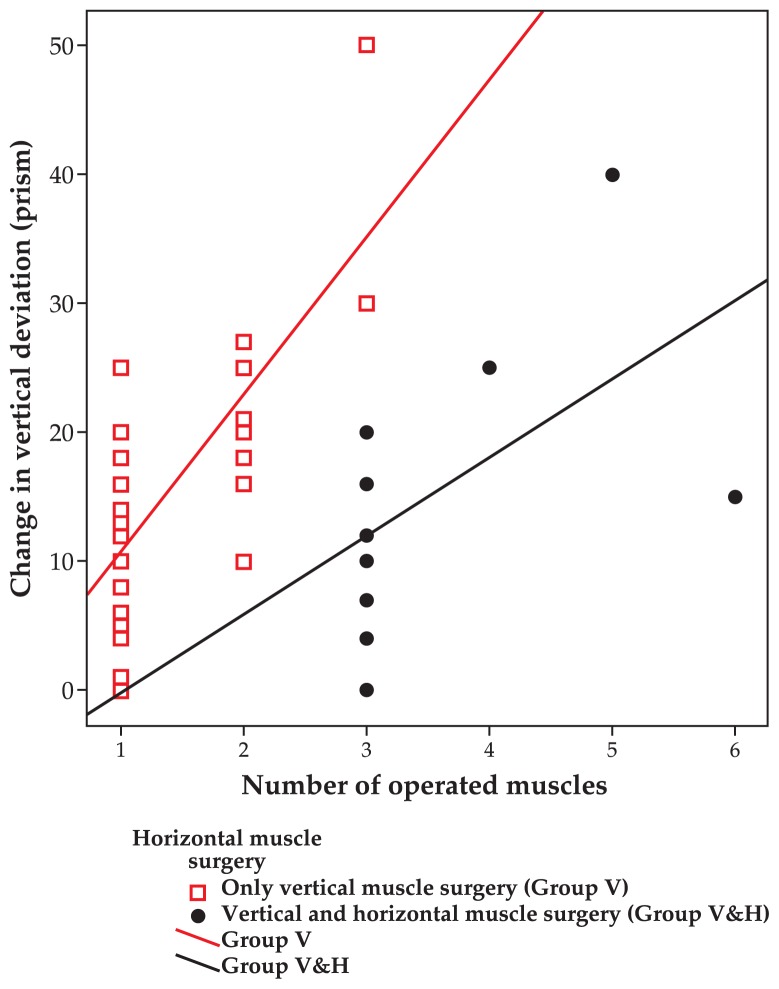
Relationship between the number of operated vertical muscles with improvement in vertical deviation with and without horizontal muscle surgery. Group V (R^2^: 0.525, P<0.001); Group V&H (R^2^: 0.294, P:0.035).

The number of operated muscles in the first surgery ranged from one muscle in 45 (61.6%) cases to 6 muscles in one (1.4%) case. The mean number of operated muscles during initial surgery was 1.7±1.1. Increasing the number of operated cyclovertical muscles increased vertical improvement which was more significant when horizontal muscles were not operated (Fig. 5, Spearman correlation).

At final follow-up, complete success including orthophoria, improved head position and reduced complaints was achieved in 75 (78.1%) patients; 16 (21.9%) cases achieved partial success including acceptable improvement in symptoms, abnormal head posture and deviation.

## DISCUSSION

The most common type of fourth nerve palsy in our series was congenital (76.7%) which is very close to the 76.9% figure reported by Ellis et al^14^ in 108 patients; the corresponding figure was 39.5% in Von Noorden’s study^1^ on 270 patients. In the Mayo Clinic study on 160 children, trauma was the most common cause of all extraocular palsies.^8^ In the study by Simons^15^ on 123 cases, trauma was also the most common cause (34%). The higher rate of congenital SO palsy in our study may be due to neglecting cosmetic problems in young children.

In our study, the most common Knapp type was Knapp III (42.5%) and the least common was Knapp type II (5.5%). In the series reported by Von Noorden and Campos,^1^ the most common was type II (31%) and the rarest was type VII (0.5%).

Simons^15^ studied 123 patients with superior oblique palsy with mean age of 31.8 years, of whom 67% were male. They operated on one muscle in 54%, two muscles in 64% and 3 muscles in 2% of their patients. The result was excellent in 60% of cases and mean deviation decreased from 14 to 4.3 PD. In our series fourth nerve palsy was also more common in male subjects, which may be secondary to the higher rate of trauma in male subjects, but our patients were younger (mean age, 19.7 years). Mean vertical deviation in our cases improved from 16.1 PD to 1.9 PD which compares favorably with the above-mentioned study and an excellent result was achieved after one surgery in 83.6% of cases in our series which was higher than the above mentioned study.

Simons^15^ performed SO tucking in 23% of his cases but only 11% of our patients with confirmed tendon laxity underwent this procedure. We believe that IO weakening is a safe and effective alternative procedure. Mean vertical effect of IO weakening in our cases was 12 PD.

Associated horizontal strabismus was present in 65.8% of patients preoperatively which improved spontaneously in 62% of subjects with exotropia and 45% of cases with esotropia after operating on cyclovertical muscles. Therefore when associated horizontal deviation is less than 15 PD it seems advisable not to operate on horizontal muscles and postpone such surgery until correction of vertical strabismus.

In summary, cosmetic problems are the most common complaint in fourth nerve palsy, the most common type of the disorder is congenital, it is most common in young male subjects, concomitant horizontal deviation is frequent and exotropia is more common than esotropia. Its surgical treatment is highly successful if tailored according to the severity of the primary deviation and addressing gazes with the most significant deviation. Surgery directed to the SO which is the main involved muscle should be reserved for cases with significant tendon laxity or when torsion is the predominant problem.

## Figures and Tables

**Table 1:** Clinical findings in superior oblique palsy

	No	%
Eye deviation	38	52.1
Diplopia	19	26.0
Amblyopia	14	19.2
Abnormal head posture	7	9.6
Facial asymmetry	5	6.8

**Table 2:** Frequency of different Knapp groups

Knapp Type	Frequency	Percent
1	10	13.7
2	4	5.5
3	31	42.5
4	9	12.3
5	10	13.7
6	9	12.3

Total	73	100
